# Extreme Heat and Behavioral Symptoms of Cognitive Decline in Older Mexican Americans

**DOI:** 10.1093/geroni/igaf122.895

**Published:** 2025-12-31

**Authors:** Phillip Cantu, Sadaf Milani

**Affiliations:** UTMB Health, The University of Texas Medical Branch, Galveston, Texas, United States; University of Texas Medical Branch, Galveston, Texas, United States

## Abstract

Extreme temperatures are associated with worse cognitive functioning among older adults. It remains unknown if these patterns are consistent for behavioral symptoms of cognitive decline. Mexican American older adults have historically lived mostly in the southwestern United States in states that are particularly vulnerable to high heat. We examined the association of extreme heat, as measured by days of heat indices above 90 degrees a year, with informant reports of behavioral symptoms of cognitive decline, as measured by the Neuropsychiatric Inventory (NPI), in a large representative sample of older Mexican Americans, the Hispanic Established Population for the Epidemiologic Study of the Elderly. Using OLS regression, we found that individuals who experienced more days of extreme heat had more reports of behavioral symptoms of cognitive impairment, net of actual cognitive functioning. Additionally, we found that the relationship between behavioral symptoms and extreme heat were stronger for people with cognitive impairment, defined as a Mini-Mental Status Examination score of less than <21. We also found that extreme heat days were associated with specific NPI symptoms including engaging in repetitive activities, losing interest in usual activities, excessive happiness (mania), low spirits (depression), and hearing voices. These results suggest that the behavioral manifestations of cognitive impairment may be related to the physical environment older adults live in. Future research should examine the extent to which the built environment and physical structures may mediate the relationship between extreme heat and behavioral symptoms of cognitive decline.

